# Supplementary material for: Exploring structural variation and gene family architecture with De Novo assemblies of 15 Medicago genomes
Source: BMC Genomics. 2017 Mar 27;18:261. doi: 10.1186/s12864-017-3654-1 (PMC5369179; doi:10.1186/s12864-017-3654-1)
Supplement: Supplementary file 1 — Supplementary figures (Figure S1-S5) described in the manuscript. (DOCX 183 kb) [file 12864_2017_3654_MOESM1_ESM.docx]

***Exploring Structural Variation and Gene Family Architecture with De Novo Assemblies of 15 Medicago Genomes***

Running head: ***De novo* assemblies of *Medicago* genomes**

**Peng Zhou^1^, Kevin A. T. Silverstein^2^, Thiruvarangan Ramaraj^3^, Joseph Guhlin^4^, Roxanne Denny^1^, Junqi Liu^5^, Andrew D. Farmer^3^, Kelly P. Steele^6^, Robert M. Stupar^5^, Jason R. Miller^7^, Peter Tiffin^4^, Joann Mudge^3^, Nevin D. Young^1,4^ ***

**Affiliations**

1 Department of Plant Pathology, University of Minnesota, St. Paul, MN, USA

2 Supercomputing Institute for Advanced Computational Research, University of Minnesota, Minneapolis, MN, USA

3 National Center for Genome Resources, Santa Fe, NM, USA

4 Department of Plant Biology, University of Minnesota, St. Paul, MN, USA

5 Department of Agronomy and Plant Genetics, University of Minnesota, St. Paul, MN, USA

6 Science and Mathematics Faculty, Arizona State University, Mesa, AZ, USA

7 J. Craig Venter Institute, Rockville, MD, USA

* Corresponding Author

**Email Addresses**

Peng Zhou: zhoux379@umn.edu

Kevin A. T. Silverstein: kats@umn.edu

Thiruvarangan Ramaraj: tr@ncgr.org

Joseph Guhlin: guhli007@umn.edu

Roxanne Denny: denny004@umn.edu

Junqi Liu: liuxx162@umn.edu

Andrew D. Farmer: adf@ncgr.org

Kelly P. Steele: Kelly.Steele@asu.edu

Jason R. Miller: jmiller@jcvi.org

Peter Tiffin: ptiffin@umn.edu

Joann Mudge: jm@ncgr.org

Nevin D. Young: neviny@umn.edu

## Abstract

**Background**

Previous studies exploring sequence variation in the model legume, *Medicago truncatula*, relied on mapping short reads to a single reference. However, read-mapping approaches are inadequate to examine large, diverse gene families or to probe variation in repeat-rich or highly divergent genome regions. *De novo* sequencing and assembly of *M. truncatula* genomes enables near-comprehensive discovery of structural variants (SVs), analysis of rapidly evolving gene families, and ultimately, construction of a pan-genome.

**Results**

Genome-wide synteny based on 15 *de novo* *M. truncatula* assemblies effectively detected different types of SVs indicating that as much as 22% of the genome is involved in large structural changes, altogether affecting 28% of gene models. A total of 63 million base pairs (Mbp) of novel sequence was discovered, expanding the reference genome space for *Medicago* by 16%. Pan-genome analysis revealed that 42% (180 Mbp) of genomic sequences is missing in one or more accession, while examination of *de novo* annotated genes identified 67% (50,700) of all ortholog groups as dispensable – estimates comparable to recent studies in rice, maize and soybean. Rapidly evolving gene families typically associated with biotic interactions and stress response were found to be enriched in the accession-specific gene pool. The nucleotide-binding site leucine-rich repeat (NBS-LRR) family, in particular, harbors the highest level of nucleotide diversity, large effect single nucleotide change, protein diversity, and presence/absence variation. However, the leucine-rich repeat (LRR) and heat shock gene families are disproportionately affected by large effect single nucleotide changes and even higher levels of copy number variation.

**Conclusions**

Analysis of multiple *M. truncatula* genomes illustrates the value of *de novo* assemblies to discover and describe structural variation, something that is often under-estimated when using read-mapping approaches. Comparisons among the *de novo* assemblies also indicate that different large gene families differ in the architecture of their structural variation.

## Background

Legumes comprise a diverse and ecologically significant plant family that serves as the second most important crop family in the world [1]. As a cool season legume, *Medicago truncatula* is closely related to important crops such as alfalfa (*Medicago sativa*), clover (*Trifolium pratense* and *T. repens*), pea (*Pisum sativum*), chickpea (*Cicer arietinum*), and *Lotus japonicas* [2,3]. *M. truncatula* was chosen as a model for studying legume biology due to its small genome size, simple diploid genetics, self-fertility, short generation time, amenability to genetic transformation and large collections of diverse ecotypes [3–5]. *M. truncatula* research has focused especially on its symbiotic relationship with rhizobia and arbuscular mycorrhizae, root development, secondary metabolism and disease resistance [3,6]. A high quality, BAC-based sequence has served as the original “reference genome” for the *Medicago* research community [7] while re-sequencing of additional accessions has enriched the pool of sequence data available [8,9].

In plants, large gene families play a crucial role in both biotic interactions and abiotic response. Some of these families are encoded by hundreds of members [10–12] organized in clusters of varying size and thought to evolve through gene duplication and birth-and-death processes [13–17]. Widely studied examples include the nucleotide-binding site, leucine-rich repeat proteins (NBS-LRRs), receptor-like kinases (RLKs), F-box proteins, leucine-rich repeat proteins (LRRs), heat shock proteins (HSPs), and protein kinases [16–20]. In *M. truncatula* and close taxonomic relatives, an additional gene family is important in symbiotic nitrogen fixation, the nodule-specific cysteine-rich peptides (NCRs), a sub-family within the larger cysteine-rich peptide (CRP) superfamily [21–24]. Legume NCRs are highly expressed in rhizobial nodules [22,24,25] where they act as plant effectors directing bacteroid differentiation [26]. NCR genes are abundant, diverse, and frequently clustered [23,24].

Previous studies of plant genomes highlighted the important role that gene families play in the architecture of structural variation (SV) (reviewed in [27]). Array-based re-sequencing of 20 *Arabidopsis* accessions indicated that 60% of NBS-LRRs, 25% of F-box, and 16% of RLKs exhibited some type of major-effect polymorphism compared with less than 10% for all expressed sequences [28]. In *Arabidopsis*, 33.3% of the NBS-LRR genes in the Columbia reference are deleted in at least one of 80 accessions compared with just 12.5% of genes in the *Arabidopsis* genome as a whole [29]. In rice, Schatz et al [30] re-sequenced three divergent genomes and found that genes containing the NB-ARC domain (signature motif of NBS-LRRs) constituted 12% of lineage-specific genes compared with just 0.35% of genes shared among all three genomes.

In contrast to earlier alignment-based (read-mapping) studies of sequence diversity, *de novo* sequencing and assembly of genomes from multiple accessions enables near-comprehensive discovery of SVs, gene family membership, and ultimately, construction of a pan-genome. Here, we describe *de novo* genome assemblies for 15 *M. truncatula* accessions, which we analyze together with the *M. truncatula* reference. We were especially interested in the level and type of SVs found in different gene families, with a focus on families associated with biotic interactions and abiotic stress. Our results illustrate how different gene families exhibit distinctly different variant architectures, including differing representation within the dispensable portion of the pan-genome.

## Results

### *De novo* assemblies have scaffold N50s > 250 kb, capturing > 90% of the *M. truncatula* gene space

Fifteen *M. truncatula* accessions were sequenced with Illumina HiSeq2000 using a combination of short and long insert paired-end libraries to an average of 120-fold coverage, then assembled using ALLPATHS-LG [31] (Figure S1, Table S1). Between 80 and 94% of each genome could be assembled into scaffolds >100 kbp, with scaffold N50s ranging from 268 kbp to 1,653 kbp and contig N50 sizes averaging around 20 kbp (Table S2). Assembled genome sizes ranged from 388 Mbp to 428 Mbp (Table S2), correlating well with cytologically derived genome size estimates (r = 0.83, P = 0.005, Figure S2). Genomes were repeat-masked with a *Medicago*-specific repeat database [32]. About 20% of each assembly was annotated as repeat, which is slightly lower than the 23% repetitive content in *Medicago* reference Mt4.0, (based on accession HM101, also known as A17) (Table S2). The *de novo* assemblies also capture 87 - 96% of unique content in the reference genome, including 90 - 96% of all Mt4.0 gene coding regions.

### Genic features in *de novo* assemblies largely resemble those of the reference

All 15 genome assemblies were annotated using Augustus [33] incorporating *ab initio* gene prediction results, RNA-Seq expression evidence from a subset of accessions as well as protein homolog support from Mt4.0 reference gene models (See Methods). Evidence-guided annotation yielded comparable numbers of coding genes (60,000 to 67,000) for each of the 15 assemblies (Table S3). On average 80-90% of predicted gene models receive support from either RNA-Seq expression or Mt4.0 syntenic homologs. The number of TE-related genes in different accessions (15,000 to 20,000, Table S3) was up to 25% lower than in the Mt4.0 reference, indicating that some *de novo* assemblies missed or collapsed repetitive sequences. A closer look at the number of TE categories suggests certain families were more likely to be missed or collapsed than others (Data file S1). Median protein length (TEs excluded) ranged from 245 to 254 amino acids – nearly equal to the estimate of 255 AAs in Mt4.0.

### Structural variants span as much as 22% of the *M. truncatula* genome

Between 92 and 96% of each assembly could be aligned with the Mt4.0 reference typically leading to ~300 Mbp of sequences in syntenic blocks where single nucleotide polymorphisms (SNPs), short InDels, and large SVs could be confidently predicted (Tables S4-S6). Global comparisons revealed long syntenic blocks intermixed with shorter, poorly aligned regions that harbor numerous structural changes (Figures 1 and 2). The pattern of synteny alignment generally reflects across-accession relationships inferred from SNP data (Figure S1), including three “outgroup” accessions (HM022, HM340 and HM324) that are typically considered separate sub-species with distinct diversity patterns compared with the remaining accessions.

Within aligned genomic regions, extensive variation including SNPs, short InDels, and large SVs were observed. Between 1.7 million (HM058) and 5.1 million (HM340) SNPs were identified in comparisons with HM101 (Mt4.0) (Table S6). As expected, SNP density correlates well with divergence from HM101 – with SNP bp^-1^ ranging from 0.63% in HM058 (closest to HM101) to 2.37% in HM340 (most distant from HM101). Estimates of nucleotide diversity (θ_π_ = 0.0073 bp^-1^) are nearly 70% higher than previous reports (θ_π_ = 0.0043 bp^-1^ based on a broader 26 accession panel) (Table S4, see Discussion) [8] . Approximately 70% of *Medicago* SNPs were found in intergenic regions, which are also distinguished by the highest level of nucleotide diversity (θ_π_ = 0.0089 bp^-1^) (Table S4). Diversity was much higher for synonymous than replacement polymorphisms in coding regions (Table S4). These findings are consistent with the expectation of stronger purifying selection acting at replacement sites, especially large-effect polymorphisms that significantly alter the protein product [34].

Beyond SNPs, we identified 500,000 to 1,500,000 short InDels (<50 bp), 27,000 - 110,000 large InDels, 49,000 - 169,000 copy number variants (CNVs), and 2,700 - 12,700 translocations. SVs were identified through a rigorous syntenic anchoring approach with each SV receiving support from synteny alignments of both flanking sequences and being free from any intra- or inter- scaffold gaps (see Methods). Nevertheless, these number may still underestimate the true level of variation given that 4 to 8% of each genome could not be covered by our synteny alignment and therefore likely to involve additional complex changes (Table S5). In count, SVs are far less numerous than single-base variants, yet each of these SV classes affects more total base pairs. Small InDels affect 3 – 10 Mbp, large insertions and deletions affect 7.5 – 30 Mbp, CNVs affect 26 – 85 Mbp, and translocations affect 3.5 – 14 Mbp (Table S6). Altogether between 7% (HM058) and 22% (HM022) of genome content is affected by at least one type of structural change (Table S6). This is consistent with findings in other systems where large variants typically affect more bases than SNPs [35,36]. Nearly equivalent numbers of small insertions versus deletions were observed in contrast to traditional read mapping-based approaches (which incorrectly predict more deletions than insertions relative to the reference sequence [37,38]). Nonetheless, large deletions and copy number losses were still 30-50% higher, even with our use of synteny-based variant discovery, indicating reduced power in detecting large insertions and copy number gains (Table S6).

To estimate the accuracy of our SV prediction, we performed PacBio sequencing on three accessions (HM034, HM056 and HM340). For each SV, the number of PacBio reads fully spanning ±500bp of the breakpoints was counted and scored as valid only if each of its breakpoints received at least five supporting PacBio reads. Based on these criteria, between 88 and 94% of all synteny-based SV calls could be validated using long read technology (Table S7). Insertion and deletion of unique (single-copy) genomic contents tended to have higher validation rates than gain or loss of repetitive genomic contents (i.e., copy number gain or loss). This is consistent with assembly quality in repetitive regions generally being lower than in unique regions. Also, SVs involving genic regions tend to have the highest validation rates compared with other genomic contexts (TEs, unknown genes, intergenic). Some of the genic SVs provide good candidates in studying gene birth-and-death processes. As an example, we identified a tandem duplication of a NBS-LRR gene in HM034 (or gene deletion in HM101) which is supported by long PacBio reads (Figure S3) Interestingly, the altered gene copy doesn’t have RNA-Seq expression, whereas all the neighboring copies do, a possible indication of pseudogene removal.

Global comparisons revealed long, conserved syntenic blocks intermixed with shorter, poorly aligned regions that harbor numerous structural changes (Figures 1 and 2). The global pattern of synteny alignment generally reflect the *Medicago* phylogeny – with three “outgroup” accessions (HM022, HM340 and HM324) that are typically considered separate sub-species showing distinct diversity pattern from the remaining accessions (Figure 1, Figure 2A). Nevertheless, peri-centromeric locations generally display increased levels of diversity (and reduced levels of synteny) due to enrichment of transposable elements (TEs) (Figure 1). In genomic regions where synteny disappears altogether, our ability to identify different variant types (i.e. SNPs, short InDels, or structural variants) also disappears. This is illustrated in Figure 2 (panels B-E) where high densities of TEs and selected gene families (RLKs, NBS-LRRs, LRRs) are associated with reduced synteny coverage and loss of power in detecting all variant types (grey areas). Non-centromeric regions with higher TE density show high level of diversity and reduced synteny (e.g., Figure 1B, Figure 2B). Like TEs, large clusters of NBS-LRRs, RLKs and LRRs lead to fragile genome architecture and higher level of diversity (Figure 2 C-E). Genomic locations of these gene family clusters are generally uncorrelated with one another, but there are notable examples they co-localize (Figure 2 C-E). In these highlighted regions, substantial clusters of NBS-LRRs, RLKs, NCRs, LRRs and F-box genes are all found within a single 1Mb segment.

### 180 Mbp is dispensable sequence out of a total pan-genome content of 430 Mbp

Sequences that could not be aligned to the Mt4.0 reference even at relaxed stringency (~80% sequence identity) were extensive across the 15 *de novo* assemblies. These sequences often exist in the form of novel insertions or complex substitutions, sometimes as separate scaffolds. After filtering potential contaminant sequences, we identified between 9 and 22 Mbp of novel segments (1.3 to 2.4 Mbp in coding regions) longer than 50 bp among the 15 *de novo* assemblies (Table S5). All-against-all alignments were made among these novel segments (See Method) and a total of 63 Mbp non-redundant novel sequences were identified, with 47% (30 Mbp) present in two or more accessions and 53% (33 Mbp) being specific to a single accession (Figure 3A).

Size curves for both pan- and core-genomes were obtained by adding one genome to the population pool at a time (Figure 3B). For this analysis, only the 13 “ingroup” accessions out of the total 16 were used, excluding the three distinct sub-species accessions (HM340, HM324, HM022). The core-genome size curve drops quickly at first, flattening once 5 accessions are added, though still slightly negative in slope even at the point where all 13 have been added. Approximately 250 Mbp sequences are shared among the 13 “ingroup” accessions representing conserved regions that presumably play core functions in all *M. truncatula* (Figure 3A). Another ~180 Mbp is missing from at least one accession (i.e., “dispensable”), reflecting the dynamic nature of genome content and prevalence of InDels and other SVs (Figure 3B). The corresponding pan-genome size curve sees steady increases each time a new genome is added, approaching 430 Mbp when all 13 accessions have been added. Indeed, fitting the observed pan-genome curve using a asymptotic regression model led to estimates for the total pan-genome size of 431 Mbp and a core-genome of 256 Mbp for *M. truncatula.*

To understand the effect of sequence variation on gene families, we annotated all *de novo* assemblies and systematically identified orthologous relationships for each gene among the 13 ingroup accessions – *i.e.*, the entire collection of ortholog groups in the population. We placed a total of 607k non-TE genes (44k to 47k per accession) into 75k ortholog groups based on sequence similarity. On average each ortholog group contained 8.1 protein sequences coming from six different accessions (see Methods, Figure 4). In addition to the 37k reference (Mt4.0 / HM101) ortholog groups, this analysis resulted in another 38k ortholog groups with no HM101 members. We identified a substantial number (25k) of accession-specific genes that were only observed in a single accession, 25.7k ortholog groups shared by 2-12 accessions, and 24k more shared among all 13 (Figure 4). Accession-specific ortholog groups numbered as few as 1,500 specific to accession HM060 and as many as 3,000 specific to HM101.

### Variation in different gene families results from differing mechanisms

Several different diversity measures were estimated for different gene families (Figure 5; Figure S4 A-D). The θ_π_ statistic, large effect SNP change, and mean protein pairwise distance are metrics that provide insights into the rates of evolution for different gene families, while the coefficient of variation (C.V.) of ortholog groups tracks the level of copy number variation (orthology vs paralogy). The gene families we examined exhibit distinctly different patterns of variation compared with the genome as a whole and among themselves (Figure 5; Figure S4). NBS-LRRs are in every aspect like TEs, showing the highest SNP diversity (θ_π_), most frequent large-effect SNP changes (premature stop codon, start codon lost, stop codon lost and splice site changes), highest mean pairwise protein distance (a proxy for all protein structural variants), enrichment in accession-specific gene content, and highest ortholog group size coefficient of variation (CNV) (Figure 5; Figure S4). LRRs and HSPs show intermediate levels of SNP diversity and pairwise protein distance, but are frequently affected by large effect SNP changes and even higher CNV (Figure 5; Figure S4). RLKs, F-box proteins and NCRs all show elevated levels of certain diversity measures, but are much less diverse then NBS-LRRs, LRRs or HSPs. Interestingly, protein kinases show high CNV despite low levels of SNP diversity and pairwise protein distance. Differences in variant architecture among gene families are illustrated in Figure 6, where the percent sequence similarity between the reference gene model and its syntenic orthologs in the other 15 accessions is shown for three example protein families (Zinc-Finger, NCRs and NBS-LRRs). Both the NCR and NBS-LRR protein families are clearly more variable than Zinc-Fingers, but NBS-LRRs exhibit more orthologs with significant sequence dissimilarities (structural variants, red color) as well as higher numbers of CNVs (white regions corresponding to missing orthologs).

We further examined these gene families to estimate their contribution to accession-specific ortholog groups (Figure S5). Most striking were TEs, 49.2% of which were accession-specific compared with just 8.3% in the core set of ortholog groups (6.0x). Likewise, LRRs (50.2% accession-specific, 10.4% core; 4.8x), NBS-LRRs (45.3% accession-specific versus 10.7% core; 4.3x), HSP70s (41.2% accession-specific versus 19.3% core; 2.1x) and protein kinases (43.6% accession-specific versus 23.4% core; 1.9x) were all over-represented in terms of accession-specific ortholog groups. By contrast, NCRs (23.8% accession-specific versus 34.1% core; 0.7x), F-box proteins (17.6% accession-specific versus 44.5% core; 0.4x) and RLKs (23.4% accession-specific versus 60% core; 0.4x) (Figure S5) all showed lower rates of representation in the accession-specific portion of the genome.

##

## Discussion

### Synteny analysis based on *de novo* assemblies effectively discovers SNPs, small InDels and large SVs

Exploring plant genome variation increasingly involves the sequencing of multiple accessions within a species. Early efforts simply aligned short reads against a reference to discover SNPs and short indels (so-called “read-mapping approach”). This includes our own earlier surveys of *M. truncatula* variation [8,9] as well as similar studies in *Arabidopsis*, maize, soybean, rice and others [39–45]. In these previous analyses, variation in very divergent or repetitive regions, as well as larger and more complex types of variation would typically have been overlooked. Recent studies have turned to *de novo* genome assembly combined with synteny comparison as a basis for exploring genome variation. In *Arabidopsis*, sequencing and assembling multiple genomes led to the discovery of 14.9 Mb Col-0 sequences missing in at least one other accession along with unprecedented proteome diversity [46]. In soybean, comparison of multiple wild relatives against the reference found that 20% of the genome and 51.4% of gene families were dispensable and also identified hundreds of lineage-specific genes as well as genes exhibiting CNVs as potential targets of selection [47]. Sequencing three divergent rice strains revealed several megabases of novel sequences specific to one strain [30]. In the present study, we deeply re-sequenced 15 *M. truncatula* accessions and used the ALLPATHS-LG algorithm to create high quality assemblies followed by synteny comparison as a basis for global variant discovery. The resulting genome assemblies had scaffold N50s >250 kb and synteny coverage >92% of the *M. truncatula* reference Mt4.0. Synteny-based estimates of θ_w_ (Watterson’s estimator of population mutation rate) suggests the level of diversity is 30% higher than original read-mapping published estimates (Table S4) [8]. Looking at θ_π_ (i.e., average number of nucleotide differences per site between two accessions), the underestimate is 70%, though this could be due, in part, to a more complete reference, deeper sequencing of the accessions used in this study, and/or population structure among the selected accessions. Examination of the syntenic blocks enabled extensive, high confidence discovery of SVs, including most large indels, CNVs and translocations. These SVs affect 7-22% of the alignable genome space for each *Medicago* accession, with large indels spanning as much as 30 Mbp per accession and CNVs affecting as much as 85 Mbp (out of a genome ~450 Mbp in total size). The values reported here provide a better estimate of genomic diversity within *M. truncatula*, allowing for divergent genomic regions to be assessed accurately and helping to resolve repetitive and variable genomic regions and gene families.

### The *Medicago* pan-genome largely resembles that of other analyzed plant species

*De novo* sequencing of multiple accessions enabled us to construct a draft pan-genome for *M. truncatula*, indicating a core genome of ~250 Mbp and a dispensable genome of ~180 Mbp (Figure 3B). Annotation of the *Medicago* *de novo* genomes followed by clustering using OrthoMCL resulted in a core set of 24,000 (non-TE) ortholog groups present in all *M. truncatula* accessions sequenced and another 50,700 (67% of the total) that are dispensable (Figure 4). As *de novo* genomes were added during the pan-genome analysis, the rate of increase declined quickly, with both the pan-genome and core-genome curves nearly flat with the last genome added. Limited novel sequence discovery would therefore be expected with the addition of further accession genomes. Indeed, our estimation suggests an asymptotic pan-genome size of 431 Mbp and core-genome of 256 Mbp (Figure 3). Similar trends have been observed in pan-genomic analyses of seven *de novo* *Glycine soja* genome [47], ten *Brassica oleracea* genomes [48], as well as a pan-transcriptome analysis 503 maize accessions [49], results that together suggest higher plant pan-genomes may generally be restricted in size. The finding that 67% of *Medicago* ortholog groups are dispensable is likewise comparable to earlier estimates of 51% in the *G. soja* analysis mentioned above [47], 73% in a study of five *Oryza* AA genomes [50], and 83% of the representative transcript assemblies (RTAs) in the pan-transcriptome analysis of maize [49]. All these values are higher, however, than an estimate of just ~20% dispensable gene families observed in the study of the *B. oleracea* pan-genome, an observation that might be attributable to their focus on cultivated genotypes [48].

Important caveats should be kept in mind when interpreting these results. Due to the incompleteness of the *de novo* *Medicago* assemblies (*i.e.*, certain portions of genome were difficult to assemble), sequences present in one assembly but absent in others could have been due to technical artifact. This would have resulted in overestimates of dispensable genome size. By contrast, the pan-genome size estimate should be more robust since it surveys novel sequences across all accessions – and it is much less likely that a given genome region would be missed in all assemblies.

### Differences in variant architecture among different gene families

Genome regions high in SVs often coincide with genome regions rich in either TEs or one of the biotic interaction and stress related gene families examined in this study (Figures 1 and 2). This is a relationship that has frequently been observed in plant genomes [30,46–48,50], but in our study, we were especially interested in the range and type of SVs found in different gene families (Figure 5, Supplemental Figure S4A-D). NBS-LRRs are the most variable and the most like TEs in their variant structure. Both NBS-LRRs and TEs exhibit frequent large-effect SNP changes, very high levels of protein diversity (mean protein distance), enrichment in the accession-specific gene content, and high levels of CNVs (C.V. of gene copy number). While LRRs and HSPs only exhibit intermediate levels of SNP diversity and protein diversity, they are frequently affected by large effect SNP changes and even higher levels of CNV. Like NBS-LRRs, these two gene families are over-represented in accession-specific gene content. By contrast, protein kinases show notably low SNP and protein diversity together with high levels of CNVs and over-representation in accession-specific content. Finally, RLKs, F-box proteins, and NCRs are all much less diverse than the other families studied here. Not surprisingly, they are also under-represented in terms of accession-specific gene content. Some of these differences make sense when considering the genome features of different gene families. For example, NBS-LRRs have long been known to include a large proportion of pseudogenes [51], a feature thought to result from the value of maintaining a reservoir of genetic diversity against future pathogen pressure. Consequently, very high levels of large-effect SNPs are to be expected. Likewise, NBS-LRRs are large, multi-module proteins, so high levels of protein diversity, often involving domain swapping, should be common [10,13–15]. By contrast, NCR genes, which are just as numerous and comparably clustered in the *M. truncatula* genome, code for expressed, short, single peptide, modular proteins [24,25,51]. Not surprisingly, NCRs are quite low in large effect SNPs.

### Limitations remain in *de novo* assemblies based on short read sequencing technology

Even with very deep re-sequencing and *de novo* assembly using the ALLPATHS-LG algorithm, important limitations remain. The contig N50 for most assemblies was only 20 kb and any of the thousands of sequencing gap potentially represents a missing SV. We also lacked the ability to discover SVs in regions without synteny to the Mt4.0 reference. Altogether, these missing regions account for 4-8% of the genome space for each *Medicago* accession. Moreover, gaps remaining in the Mt4.0 reference reduce its effectiveness as a framework for SV discovery. These factors all presumably result in missed SV calls. Nevertheless, the SVs we did predict could largely be validated. By comparing SVs discovered in the ALLPATHS assemblies of three *M. truncatula* accessions to (a minimum of five) long uninterrupted reads coming from PacBio sequencing, we confirmed 88-94% of SV predictions from our synteny analysis. As more PacBio and other long read technologies are used to resequence and assemble genomes, fewer gaps will remain and analyses of SVs, dynamic gene families, and pan-genomes will become more complete and accurate.

**Conclusions**

Analysis of multiple *M. truncatula* genomes illustrates the value of *de novo* assemblies to discover and describe structural variation, something that is often under-estimated when using read-mapping approaches. Comparisons among the *de novo* assemblies also indicate that different large gene families differ in the architecture of their structural variation.

## Methods

### Plant material

Fifteen *M. truncatula* accessions from geographically distinct populations (Figure S1) broadly spanning the entire *Medicago* range were chosen for deep sequencing and *de novo* assembly. These accessions were chosen for both biological interest and to facilitate evaluation of assemblies. In particular, three accessions were selected from the A17 clade, nine were selected from the France-Italy clade, and three were selected from more distantly related clades [52]. While most analyses were done on all 16 accessions including the reference HM101, some statistics sensitive to population structure were derived from a subset of 13 accessions (three distant accessions were excluded), which we refer to as “ingroup” accessions. Each accession was self-fertilized for three or more generations before growing seedlings for DNA extraction. Cloning and sequencing grade DNA was extracted from a pool of ~30 day old dark-grown seedlings by Amplicon Express (Pullman, WA) through Ultra Clean BAC Clone Preparation followed by a CTAB liquid DNA preparation [53].

### Sequencing and genome assembly

Library preparation, sequencing and assembly were performed at the National Center for Genome Resources (NCGR) in Santa Fe, NM. DNA sequencing was performed using Illumina HiSeq 2000 instruments. For each accession, one Short Insert Paired End (SIPE) library and 1 - 2 Long Insert Paired End (LIPE) libraries were created following the ALLPATHS-LG assembler [31].The SIPE library consisted of fragments of ~300 nucleotides (180 nucleotides plus adapters) while LIPE libraries consisted of either a 5 kb Illumina or 9 kb Nextera library. The ALLPATHS-LG assembly algorithm (version 49962) [31] was run on a linux server with default parameters to complete the assemblies.

### Functional annotation

AUGUSTUS [33] was used to make *ab initio* gene predictions for each assembly using both RNA-Seq expression evidence and *M. truncatula* HM101 reference sequence (Mt4.0) [7] homology evidence. RNA-Seq data came from transcript sequencing of four diverse accessions, HM034, HM056, HM101 and HM340. Reads from HM034, HM056 and HM340 were directly mapped to their *de novo* assemblies using Tophat [54] to generate intron hints for AUGUSTUS. For the remaining 12 accessions, RNA-Seq reads from the closest available accession were mapped to the corresponding assembly to generate intron hints. Predicted protein sequences were scanned for PFAM domains (Pfam-A.hmm) [55] using HMMER [56] and processed using custom scripts. Domain categories were then assigned according to the most significant Pfam hits. Among the resulting Pfam domains, 160 were associated with transposable elements and grouped into a large “TE" category. NBS-LRR and RLK genes were scanned using sub-family alignments from previous work [57] with 37 NBS-LRR sub-family identifiers (TNL0100-TNL0850, CNL0100-CNL1600) and 35 RLK sub-family identifiers (LRR_I-LRR_XIII, RLCK_I-RLCK_XI) created in consistent with previous research. NCRs and the broader CRP super-family were annotated by running the SPADA pipeline [58] with group identifiers exactly following previous literature [23]: sub-family CRP0000-CRP1030 representing defensing-like genes (DEFLs), CRP1040-CRP1530 representing NCRs, and CRP1600-CRP6250 representing other types of CRPs.

### Flow cytometry genome size estimates for *Medicago* accessions

Nine accessions (HM004, HM005, HM006, HM029, HM030, HM034, HM056, HM101 and HM324) were examined for cytological genome size. Seeds of known size standards were also obtained from Dolezel [59]. Seedlings were grown in chambers under identical light and humidity conditions, then leaf nuclei were prepared following the procedure of [59] and analyzed on a BD FACS-Calibur flow cytometer at the Bio-Design Institute, Arizona State University. Mean DNA content was based on 15,000 nuclei, with peak means identified using Cell-Quest software (Becton Dickson). Each plant accession was sampled 3 or more times on different days. Correlation analysis was then done between these cytological estimates of genome size and assembled genome sizes to make Figure S2.

### Comparative genomics analysis

Each *de novo* assembly was first aligned to the HM101 reference (i.e., Mt4.0) using BLAT [60]. Unaligned sequences (query sequences with no hit to the reference) were extracted and aligned a second time because BLAT tended to over-extend gap length when it encountered stretches of ‘N’s (i.e., assembly gap) in the target sequence. The resulting alignments were merged, fixed (removing non-syntenic or overlapping alignment blocks), and cleaned (removing alignment blocks containing assembly gaps). BLAT Chain/Net tools were then used to obtain a single coverage best alignment net in the target genome (HM101) as well as a reciprocal-best alignment net between genomes. Finally, genome-wide synteny blocks were built for each *de novo* assembly (against HM101), enabling downstream analyses including variant calling, novel sequence identification, and ortholog detection.

Based on synteny blocks generated, we identified SNPs, short InDels (alignment gaps ≤ 50 bases), and different types of SVs including large deletions, insertions, translocations and copy number gains and losses. SVs were identified in a rigorous syntenic anchoring approach: scaffolds were first aligned to and anchored on the HM101 reference genome, genome-wide synteny blocks were then built for each *de novo* assembly (against HM101). SVs were then called only in these well-built synteny blocks, with each SV (insertion, deletion or translocation) receiving support from both flanking sequence alignments. Variants, including large SVs, from the 15 accessions were merged to a single VCF file using Bcftools [61]. Since variants were called independently in different accessions, the merging process resulted in missing data for any variant/accession combinations where the variant was not called in that accession. Custom scripts were run to impute "reference genotype" for these variant/accession combinations whenever the underlying synteny alignment supports the non-variant (i.e., reference) allele call. We then partitioned the reference genome into 1-Mbp sliding windows to calculate gene density, TE density, selected gene family density, as well as pairwise nucleotide diversity (θ_π_) for SNPs, short InDels and SVs within each window.

### Pan-genome construction and identification of accession-specific genes

Based on pairwise genome comparison of each *de novo* assembly against the reference (HM101), we obtained a raw set of novel sequences (present in *de novo* assembly but absent in HM101) by subtracting all aligned regions from the gap-removed assembly. Low-complexity sequences and short tandem repeats were scanned and removed using Dustmasker and Tandem Repeat Finder [62,63]. Potential contaminant sequences (best hit in non-plant species) were filtered by BLASTing [64] against NCBI Nucleotide (nr/nt) database. Contamination removal was done after pairwise comparison with the HM101 reference based on the logic that everything that aligns to HM101 should be of plant origin and free of contaminant, so it was only necessary to scan the sequences that do not align to HM101 - i.e., novel sequences. Novel sequences (longer than 50 bp) from 12 accessions (13 “ingroup” accessions excluding HM101) were pooled and aligned using Para-Mugsy [65]. The resulting alignments were parsed to determine how each segment was shared among accessions – private to one accession or shared by multiple. We then constructed a pan-genome that included the HM101 reference as backbone plus all non-redundant novel segments identified in the other accessions. We further derived genome size curves by adding one *de novo* assembly to the pool at a time and calculating the size of shared genomic regions (core-genome) and the size of total non-redundant sequences (pan-genome). The pan- and core-genome size size curves were fitted using the asymptotic regression model y = b0 + b1*(1-exp(-exp(lrc) * x)) [66]. The model was fitted using means.

Accession-specific genomic segments were extracted from Para-Mugsy alignments mentioned above. Genes with more than 50% CDS locating in these regions were selected to make the accession-specific gene set. Pfam analysis and functional enrichment were then performed on this accession-specific gene list.

### Protein ortholog group analysis and comparisons

Protein sequences from all 16 accessions (1,028,566 total genes) were pooled to construct ortholog groups using OrthoMCL [67]. This resulted in 150k ortholog groups with an average of 6 genes per group. Further analysis only focused on non-TE genes in 13 “ingroup” accessions since the three distant accessions (HM340, HM324, HM022) tend to introduce extra ortholog group due to high divergence. Ortholog groups could contain from 0 to any number of protein sequences from any one accession. A total of 607k non-TE genes from 13 ingroup accessions were grouped into 75k ortholog groups. Grouping of protein sequences was based on BlastP significance so the actual sequence similarities within groups vary – but typically above 70% identity threshold (i.e., pairwise protein distance less than 0.3). On average, each ortholog group contains 8.1 protein sequences, but from only 6.7 different accessions. For each group a functional category was assigned based on Pfam annotation of all group members. Ortholog groups were also binned based on the number of accessions contributing to them: from 1 (accession-specific) to 13 (present in all ingroup accessions, i.e., “core” ortholog groups).

### Diversity of different gene families

SNPs were called based on pairwise genome comparisons of each accession against HM101. SNP-based nucleotide diversity (θ_π_) was estimated for coding regions of each gene and the distribution of θ_π_ for different gene families was obtained. To account for poorly covered regions, only genes where ≥80% of the CDS regions were covered in at least 10 out of the 13 accessions were retained. Functional effects of SNPs in genic regions were determined using snpEff [68], and the proportion of genes with large effect SNP changes (e.g., gain or loss of stop codon) in each gene family was calculated.

In addition to SNPs, we identified a large number of small InDels and large SVs inside/overlapping genic regions. Since these types of variants often lead to frame-shift, splice-site change, exon skipping, domain swapping or other gene structural changes, we decided to use protein sequence distance as a measure to quantify the functional impact of SVs. Since the OrthoMCL-defined ortholog groups do not explicitly define one-to-one orthologous relationship among accessions, we used synteny alignment information and derived a smaller set of syntenic ortholog groups with one-to-one relationship among accessions. Filtering was done requiring syntenic orthologs be present in ≥10 accessions (i.e., missing data in less than 3 accessions) for each group. We then did multiple-sequence alignment for each syntenic ortholog group, calculated mean pairwise protein distance (MPPD), and characterized the distribution of MPPDs for different gene family categories (Pfam domains).

To assess the level of copy number variation (CNV) for different gene families, we grouped protein sequences from 13 accessions into ortholog groups using OrthoMCL (see previous section). Pfam category of each ortholog group was assigned by the most abundant category among group members. Members in each ortholog group were treated as copies of a common ancestor, thus enabling quantification of gene copy number variation among accessions. In practice, we calculated the coefficient of variation (C.V.) of gene copy number among accessions for each ortholog group and summarized its distribution for different gene families.

**Validation of SVs using PacBio long reads**

We performed PacBio sequencing on three accessions (HM034, HM056 and HM340) to validate the breakpoints of identified structural variants. Each accession was sequenced to 14-20 fold coverage using either P4C2 or P5C3 chemistry. The average read length was 4-7 Kbp. PacBio reads were first mapped to the corresponding ALLPATHS assembly using BLASR [69]. For each SV, the number of PacBio reads fully spanning ±500bp of the breakpoints were counted. We consider an SV to be “validated” only if each of its breakpoints received at least five such PacBio reads support.

### Declarations

## Acknowledgments

## We thank the University of Minnesota Supercomputing Institute for computational infrastructure, storage, and systems administrative support.

**Funding**

This research was funded by National Science Foundation Grant 1237993 to NDY, PT, KATS, RMS, JRM and JM. The funding source was not involved in the design of the study, collection, analysis, interpretation of data, or in the writing of the manuscript.

### Availability of data and materials

Illumina and PacBio reads data from this article can be found in the NCBI Sequence Read Archive (SRA) under accession number PRJNA256006. RNA-Seq reads can be found under SRA accession number SRP077692. Genome assembly sequences, SNP genotype files are available for download from the *Medicago* Hapmap project website (<http://www.medicagohapmap.org/downloads/assemblies>).

## Author Contributions

Conceived and designed experiments: KATS, RMS, PT, JRM, JM, NDY. Performed experiments: PZ, TR, RD, JL, KPS. Analyzed data: PZ, KATS, TR, JG, JL, ADF, KPS, RMS, PT, JRM, JM, NDY. Wrote paper: PZ, KATS, NDY. Collected and processed sequence data: PZ, KATS, TR, JG, JL, ADF, RMS, JRM, JM.

**Competing interests**

The authors declare that they have no competing interests.

**Consent for publication**

Not applicable.

**Ethics approval and consent to participate**

*Medicago* germplasm resources (seed) were obtained and used, with permission, from Jean-Marie Prosperi at Unité mixte de recherche / Amélioration génétique et adaptation des plantes méditerranéennes et tropicales (UMR-AGAP) at INRA-Montpellier, France.

## Additional Files

**Additional file 1**: Supplementary figures (Figure S1-S5) described in the manuscript.

**Additional file 2**: Supplementary tables (Table S1-S7) described in the manuscript.

**Additional file 3**: Supporting data file S1 (Excel spreadsheet listing the member counts of different gene families including all NBS-LRR, NCR, RLK and TE subfamilies, that are predicted in 15 *de novo* assemblies).

## References

1. Graham PH. Legumes: Importance and Constraints to Greater Use. Plant Physiol. 2003;131:872–7.

2. Lavin M, Herendeen P, Wojciechowski M. Evolutionary Rates Analysis of Leguminosae Implicates a Rapid Diversification of Lineages during the Tertiary. Syst. Biol. 2005;54:575–94.

3. Young ND, Udvardi M. Translating Medicago truncatula genomics to crop legumes. Curr. Opin. Plant Biol. 2009;12:193–201.

4. Ronfort J, Bataillon T, Santoni S, Delalande M, David JL, Prosperi J-M. Microsatellite diversity and broad scale geographic structure in a model legume: building a set of nested core collection for studying naturally occurring variation in Medicago truncatula. BMC Plant Biol. 2006;6:28.

5. Tadege M, Wen J, He J, Tu H, Kwak Y, Eschstruth A, et al. Large-scale insertional mutagenesis using the Tnt1 retrotransposon in the model legume Medicago truncatula. Plant J. 2008;54:335–47.

6. Oldroyd GED, Downie JA. Coordinating Nodule Morphogenesis with Rhizobial Infection in Legumes. Annu. Rev. Plant Biol. 2008;59:519–46.

7. Tang H, Krishnakumar V, Bidwell S, Rosen B, Chan A, Zhou S, et al. An improved genome release (version Mt4.0) for the model legume Medicago truncatula. BMC Genomics. 2014;15:312.

8. Branca A, Paape TD, Zhou P, Briskine R, Farmer a. D, Mudge J, et al. Whole-genome nucleotide diversity, recombination, and linkage disequilibrium in the model legume Medicago truncatula. Proc. Natl. Acad. Sci. 2011;108:E864–70.

9. Stanton-Geddes J, Paape T, Epstein B, Briskine R, Yoder J, Mudge J, et al. Candidate Genes and Genetic Architecture of Symbiotic and Agronomic Traits Revealed by Whole-Genome, Sequence-Based Association Genetics in Medicago truncatula. PLoS One. 2013;8:e65688.

10. Meyers BC. Genome-Wide Analysis of NBS-LRR-Encoding Genes in Arabidopsis. Plant Cell. 2003;15:809–34.

11. Shiu S-H, Bleecker a B. Receptor-like kinases from Arabidopsis form a monophyletic gene family related to animal receptor kinases. Proc. Natl. Acad. Sci. 2001;98:10763–8.

12. Kuroda H. Classification and Expression Analysis of Arabidopsis F-Box-Containing Protein Genes. Plant Cell Physiol. 2002;43:1073–85.

13. Michelmore RW, Meyers BC. Clusters of resistance genes in plants evolve by divergent selection and a birth-and-death process. Genome Res. 1998;8:1113–30.

14. Richly E, Kurth J, Leister D. Mode of Amplification and Reorganization of Resistance Genes During Recent Arabidopsis thaliana Evolution. Mol. Biol. Evol. 2002;19:76–84.

15. Leister D. Tandem and segmental gene duplication and recombination in the evolution of plant disease resistance genes. Trends Genet. 2004;20:116–22.

16. Shiu S-H, Bleecker AB. Expansion of the Receptor-Like Kinase/Pelle Gene Family and Receptor-Like Proteins in Arabidopsis. Plant Physiol. 2003;132:530–43.

17. Xu G, Ma H, Nei M, Kong H. Evolution of F-box genes in plants: Different modes of sequence divergence and their relationships with functional diversification. Proc. Natl. Acad. Sci. 2009;106:835–40.

18. Meyers BC, Dickerman AW, Michelmore RW, Sivaramakrishnan S, Sobral BW, Young ND. Plant disease resistance genes encode members of an ancient and diverse protein family within the nucleotide-binding superfamily. Plant J. 1999;20:317–32.

19. Padmanabhan M, Cournoyer P, Dinesh-Kumar SP. The leucine-rich repeat domain in plant innate immunity: A wealth of possibilities. Cell. Microbiol. 2009. p. 191–8.

20. Sung D, Kaplan F, Guy CL. Plant Hsp70 molecular chaperones: protein structure , gene family , expression and function. Physiol. Plant. 2001;113:443–51.

21. Graham M a. Computational Identification and Characterization of Novel Genes from Legumes. Plant Physiol. 2004;135:1179–97.

22. Silverstein KAT. Genome Organization of More Than 300 Defensin-Like Genes in Arabidopsis. Plant Physiol. 2005;138:600–10.

23. Silverstein K a. T, Moskal W a., Wu HC, Underwood B a., Graham M a., Town CD, et al. Small cysteine-rich peptides resembling antimicrobial peptides have been under-predicted in plants. Plant J. 2007;51:262–80.

24. Alunni B, Kevei Z, Redondo-Nieto M, Kondorosi A, Mergaert P, Kondorosi E. Genomic Organization and Evolutionary Insights on GRP and NCR Genes, Two Large Nodule-Specific Gene Families in Medicago truncatula. Mol. Plant-Microbe Interact. 2007;20:1138–48.

25. Mergaert P. A Novel Family in Medicago truncatula Consisting of More Than 300 Nodule-Specific Genes Coding for Small, Secreted Polypeptides with Conserved Cysteine Motifs. Plant Physiol. 2003;132:161–73.

26. Farkas A, Maroti G, Durg H, Gyorgypal Z, Lima RM, Medzihradszky KF, et al. Medicago truncatula symbiotic peptide NCR247 contributes to bacteroid differentiation through multiple mechanisms. Proc. Natl. Acad. Sci. 2014;111:5183–8.

27. Young ND, Zhou P, Silverstein KAT. Exploring structural variants in environmentally sensitive gene families. Curr. Opin. Plant Biol. 2016. p. 19–24.

28. Clark RM, Schweikert G, Toomajian C, Ossowski S, Zeller G, Shinn P, et al. Common Sequence Polymorphisms Shaping Genetic Diversity in Arabidopsis thaliana. Science. 2007;317:338–42.

29. Cao J, Schneeberger K, Ossowski S, Günther T, Bender S, Fitz J, et al. Whole-genome sequencing of multiple Arabidopsis thaliana populations. Nat. Genet. 2011;43:956–63.

30. Schatz MC, Maron LG, Stein JC, Wences A, Gurtowski J, Biggers E, et al. Whole genome de novo assemblies of three divergent strains of rice, Oryza sativa, document novel gene space of aus and indica. Genome Biol. 2014;15:506.

31. Gnerre S, MacCallum I, Przybylski D, Ribeiro FJ, Burton JN, Walker BJ, et al. High-quality draft assemblies of mammalian genomes from massively parallel sequence data. Proc. Natl. Acad. Sci. 2011;108:1513–8.

32. Smit A, Hubley R, Green P. RepeatMasker Open-4.0. 2013-2015 . http://www.repeatmasker.org. 2013.

33. Stanke M, Stanke M, Waack S, Waack S. Gene prediction with a hidden Markov model and a new intron submodel. Bioinformatics. 2003. p. 0.

34. Nielsen R. Molecular Signatures of Natural Selection. Annu. Rev. Genet. 2005;39:197–218.

35. Conrad DF, Pinto D, Redon R, Feuk L, Gokcumen O, Zhang Y, et al. Origins and functional impact of copy number variation in the human genome. Nature. 2010;464:704–12.

36. Redon R, Ishikawa S, Fitch KR, Feuk L, Perry GH, Andrews TD, et al. Global variation in copy number in the human genome. Nature. 2006;444:444–54.

37. Ye K, Schulz MH, Long Q, Apweiler R, Ning Z. Pindel: A pattern growth approach to detect break points of large deletions and medium sized insertions from paired-end short reads. Bioinformatics. 2009;25:2865–71.

38. Li S, Li R, Li H, Lu J, Li Y, Bolund L, et al. SOAPindel: Efficient identification of indels from short paired reads. Genome Res. 2013;23:195–200.

39. Gore M a., Chia J-M, Elshire RJ, Sun Q, Ersoz ES, Hurwitz BL, et al. A First-Generation Haplotype Map of Maize. Science. 2009;326:1115–7.

40. Lam H-M, Xu X, Liu X, Chen W, Yang G, Wong F-L, et al. Resequencing of 31 wild and cultivated soybean genomes identifies patterns of genetic diversity and selection. Nat. Genet. 2010;42:1053–9.

41. Zheng L-Y, Guo X-S, He B, Sun L-J, Peng Y, Dong S-S, et al. Genome-wide patterns of genetic variation in sweet and grain sorghum (Sorghum bicolor). Genome Biol. 2011;12:R114.

42. Chia J-M, Song C, Bradbury PJ, Costich D, de Leon N, Doebley J, et al. Maize HapMap2 identifies extant variation from a genome in flux. Nat. Genet. 2012;44:803–7.

43. Huang X, Kurata N, Wei X, Wang Z-XX, Wang A, Zhao Q, et al. A map of rice genome variation reveals the origin of cultivated rice. Nature. 2012;490:497–501.

44. Gordon SP, Priest H, Des Marais DL, Schackwitz W, Figueroa M, Martin J, et al. Genome diversity in *Brachypodium distachyon:* deep sequencing of highly diverse inbred lines. Plant J. 2014;79:361–74.

45. Xu X, Liu X, Ge S, Jensen JJDJJDJ, Hu F, Li X, et al. Resequencing 50 accessions of cultivated and wild rice yields markers for identifying agronomically important genes. Nat. Biotechnol. 2012;30:105–11.

46. Gan X, Stegle O, Behr J, Steffen JG, Drewe P, Hildebrand KL, et al. Multiple reference genomes and transcriptomes for Arabidopsis thaliana. Nature. 2011;477:419–23.

47. Li Y, Zhou G, Ma J, Jiang W, Jin L, Zhang Z, et al. De novo assembly of soybean wild relatives for pan-genome analysis of diversity and agronomic traits. Nat. Biotechnol. 2014;32:1045–52.

48. Golicz AA, Bayer PE, Barker GC, Edger PP, Kim H, Martinez PA, et al. The pangenome of an agronomically important crop plant Brassica oleracea. Nat. Commun. 2016;7:13390.

49. Hirsch CN, Foerster JM, Johnson JM, Sekhon RS, Muttoni G, Vaillancourt B, et al. Insights into the Maize Pan-Genome and Pan-Transcriptome. Plant Cell. 2014;26:121–35.

50. Zhang Q-J, Zhu T, Xia E-H, Shi C, Liu Y-L, Zhang Y, et al. Rapid diversification of five Oryza AA genomes associated with rice adaptation. Proc. Natl. Acad. Sci. 2014;111:E4954–62.

51. Marone D, Russo M, Laidò G, De Leonardis A, Mastrangelo A. Plant Nucleotide Binding Site–Leucine-Rich Repeat (NBS-LRR) Genes: Active Guardians in Host Defense Responses. Int. J. Mol. Sci. 2013;14:7302–26.

52. Yoder JB, Briskine R, Mudge J, Farmer a., Paape T, Steele K, et al. Phylogenetic Signal Variation in the Genomes of Medicago (Fabaceae). Syst. Biol. 2013;62:424–38.

53. Murray MG, Thompson WF. Rapid isolation of high molecular weight plant DNA. Nucleic Acids Res. 1980;8:4321–6.

54. Trapnell C, Pachter L, Salzberg SL. TopHat: discovering splice junctions with RNA-Seq. Bioinformatics. 2009;25:1105–11.

55. Finn RD, Bateman A, Clements J, Coggill P, Eberhardt RY, Eddy SR, et al. Pfam: the protein families database. Nucleic Acids Res. 2014;42:D222–30.

56. Eddy SR. Accelerated Profile HMM Searches. PLoS Comput. Biol. 2011;7:e1002195.

57. Ameline-Torregrosa C, Wang B-B, O’Bleness MS, Deshpande S, Zhu H, Roe B, et al. Identification and Characterization of Nucleotide-Binding Site-Leucine-Rich Repeat Genes in the Model Plant Medicago truncatula. Plant Physiol. 2007;146:5–21.

58. Zhou P, Silverstein KA, Gao L, Walton JD, Nallu S, Guhlin J, et al. Detecting small plant peptides using SPADA (Small Peptide Alignment Discovery Application). BMC Bioinformatics. 2013;14:335.

59. DOLEZEL J. Plant DNA Flow Cytometry and Estimation of Nuclear Genome Size. Ann. Bot. 2005;95:99–110.

60. Kent WJ. BLAT - The BLAST-like alignment tool. Genome Res. 2002;12:656–64.

61. Li H, Handsaker B, Wysoker A, Fennell T, Ruan J, Homer N, et al. The Sequence Alignment/Map format and SAMtools. Bioinformatics. 2009;25:2078–9.

62. Camacho C, Coulouris G, Avagyan V, Ma N, Papadopoulos J, Bealer K, et al. BLAST plus : architecture and applications. BMC Bioinformatics. 2009;10.

63. Benson G. Tandem repeats finder: a program to analyze DNA sequences. Nucleic Acids Res. 1999;27:573–80.

64. Altschul SF, Gish W, Miller W, Myers EW, Lipman DJ. Basic local alignment search tool. J. Mol. Biol. 1990;215:403–10.

65. Angiuoli S V., Salzberg SL. Mugsy: Fast multiple alignment of closely related whole genomes. Bioinformatics. 2011;27:334–42.

66. R Development Core Team. R: A Language and Environment for Statistical Computing. R Found. Stat. Comput. Vienna Austria. 2016;0:{ISBN} 3-900051-07-0.

67. Li L. OrthoMCL: Identification of Ortholog Groups for Eukaryotic Genomes. Genome Res. 2003;13:2178–89.

68. Cingolani P, Platts A, Wang LL, Coon M, Nguyen T, Wang L, et al. A program for annotating and predicting the effects of single nucleotide polymorphisms, SnpEff: SNPs in the genome of Drosophila melanogaster strain w 1118; iso-2; iso-3. Fly. 2012;6:80–92.

69. Chaisson MJ, Tesler G. Mapping single molecule sequencing reads using basic local alignment with successive refinement (BLASR): application and theory. BMC Bioinformatics. 2012;13:238.

**List of Abbreviations**

**AAs**: amino acids **CDS**: Coding sequence **CNVs**: Copy number variants **CRPs**: Cysteine-rich peptides **HSPs**: Heat shock proteins **LIPE**: Long insert paired end **LRR:** Luecine-rich repeat **Mbp**: Million base pairs **NBS-LRR**: Nucleotide-binding site leucine-rich repeat **NCRs**: nodule-specific cysteine-rich peptides **RLKs**: Receptor-like kinases **SIPE**: Short insert paired end **SNPs:** Single nucleotide polymorphisms **SVs**: Structural variants **TEs**: Transposable elements **VCF**: Variant call format

## Figures

**Figure 1. Heatmap showing percent covered by synteny alignment for each 1Mb window in 15 *de novo* *M. truncatula* assemblies (Upper 15 tracks), reference gap position (‘Gaps’), percent bases covered by synteny blocks in at least 10 out 13 accessions (‘Coverage’), nucleotide diversity (θπ) for SNPs (‘Pi_SNP’), short InDels (< 50bp, ‘Pi_InDel’) and large SVs (>= 50bp, ‘Pi_SV’), as well as gene density of different categories (TE, NBS-LRR, RLK, NCR, LRR and F-boxes).**

Nucleotide diversity (θπ) estimates were calculated using only 13 “ingroup” *M. truncatula* accessions.

**Figure 2. Zoom-in view of five 1-Mb regions (A-E) selected from Figure 1.**

Upper 15 tracks show percentage covered by synteny alignment for each 50kb window (column) in 15 *M. truncatula* assemblies. Bottom tracks show reference gap position (‘Gaps’), percent bases covered by synteny blocks in at least 10 out 13 accessions (‘Coverage’), nucleotide diversity (θπ) for SNPs (‘Pi_SNP’), short InDels (< 50bp, ‘Pi_InDel’) and large SVs (>= 50bp, ‘Pi_SV’), as well as gene density of different categories (TE, NBS-LRR, RLK, NCR, LRR and F-boxes) in relative scale (minimum to maximum spaced equally in grayscale within each panel) with grey columns representing missing data due to lack of synteny coverage. Starting poisition for each region is provided at the bottom (*e.g.,* chr7:28Mb, indicating that a 1 Mb region beginning at position 28,000,001 on chromosome 7 is displayed).

**Figure 3. Sharing status of the *Medicago* pan-genome (A) and the pan-genome size curve (B).**

**Figure 4. Sharing status of *Medicago* protein ortholog groups.**

**Figure 5. Diversity estimates of different gene families: (A) SNP-based nucleotide diversity (i.e., θ_π_), (B) proportion members affected by different types of large-effect SNPs, (C) mean pairwise protein distance for syntenic ortholog groups and (D) coefficient of variation (CV) of gene copy number in each ortholog group (i.e., an estimate of copy number variation) among accessions.**

Numbers in parenthesis reflect: (A) & (B) number of genes where ≥80% of the CDS regions were covered in at least 10 out of the 13 accessions; (C) number of syntenic ortholog groups where syntenic orthologs were present in ≥10 accessions (i.e., missing data in less than 3 accessions); (D) number of OrthoMCL-defined ortholog groups based entirely on protein sequence similarity.

**Figure 6. Sequence similarity of selected gene families in 15 *Medicago* accessions: (A) Zinc-Finger domain, (B) NCRs and (C) NBS-LRRs.**

Each cells in the score matrix indicates percent sequence similarity (1-100) between an HM101 gene and its syntenic ortholog from one of the 15 accessions. Blank (white) cells indicate missing data.

## Additional files

Additional file 1: Supplementary figures (Figure S1-S5) described in the manuscript.

Additional file 2: Supplementary tables (Table S1-S7) described in the manuscript.

Additional file 3: Excel spreadsheet listing the member counts of different gene families including all NBS-LRR, NCR, RLK and TE subfamilies, that are predicted in 15 *de novo* assemblies.
