# Supplementary material for: Exploring structural variation and gene family architecture with De Novo assemblies of 15 Medicago genomes
Source: BMC Genomics. 2017 Mar 27;18:261. doi: 10.1186/s12864-017-3654-1 (PMC5369179; doi:10.1186/s12864-017-3654-1)
Supplement: Supplementary file 2 — Supplementary tables (Table S1-S7) described in the manuscript. (DOCX 62 kb) [file 12864_2017_3654_MOESM2_ESM.docx]

Table S1. Sequencing statistics of 15 *M. truncatula* accessions.

Table S2. *De novo* assembly statistics of 15 *M. truncatula* accessions.

Table S3. Functional annotation statistics of 15 *M. truncatula* accessions.

Table S4. Coverage and diversity statistics by nucleotide class.

Table S5. Assembly comparison (with Mt4.0) statistics and novel sequences identified in 15 *M. truncatula* accessions.

Table S6. Variants identified in 15 *M. truncatula* accessions by count (A) and affected base pairs (B).

Table S7. SV validation statistics by different SV types and by genomic context.

Table S1. Sequencing statistics of 15 *M. truncatula* accessions.

|  | **Population of Origin** | **Country of Origin** | **Category** | **SIPE (Gb)** | **LIPE (Gb)** | **SIPE detail^@^** | **LIPE detail^&^** | **Coverage (fold)^*^** |
| --- | --- | --- | --- | --- | --- | --- | --- | --- |
| **HM004** | DZA045-6 | Algeria | CC8 | 25.0 | 39.2 | ~50X KAPA^#^ | ~45X Original LIPE, 34X Nextera | 128.5 |
| **HM010** | SA24714 | Italy | CC8 | 20.4 | 37.7 | ~41X KAPA | ~31X Original LIPE, ~44X Nextera | 116.3 |
| **HM022** | TN3.23 |  | RIL Parent | 24.5 | 27.9 | ~49X KAPA | ~50X Nextera | 104.9 |
| **HM023** | TN6.18 |  | RIL Parent | 24.2 | 41.4 | ~48X KAPA | ~53X Nextera | 131.3 |
| **HM034** | F20089-B | France, Corsica | CC32 | 27.1 | 32.6 | ~54X KAPA | ~65X Nextera | 119.5 |
| **HM050** | DZA058-J | Algeria | CC64 | 20.4 | 34.2 | ~41X KAPA | ~27X original LIPE, ~41X Nextera | 109.2 |
| **HM056** | ESP074-A | Spain | CC64 | 78.6 | 56.8 | ~109X TruSeq v3, ~49X KAPA | ~51X Nextera; ~33X Illumina LIPE (2-5 kb Mate Pair Sample Prep) | 270.8 |
| **HM058** | ESP163-E | Spain | CC64 | 32.2 | 35.3 | ~64X KAPA | ~45X Nextera | 135.0 |
| **HM060** | F20015-10 | France, Corsica | CC64 | 24.3 | 24.1 | ~49X KAPA | ~47X Nextera | 96.9 |
| **HM095** | F20025-4 | France, Corsica | CC96 | 26.6 | 29.7 | ~53X KAPA | ~58X Nextera | 112.5 |
| **HM125** | ESP175-A | Spain | CC144 | 21.8 | 24.0 | ~44X KAPA | ~48X Nextera | 91.6 |
| **HM129** | F20069-A | France, Corsica | CC144 | 24.8 | 22.2 | ~50X KAPA | ~44X Nextera | 93.9 |
| **HM185** | F20081-A | France, Corsica | CC192 | 21.2 | 28.6 | ~42X KAPA | ~57X Nextera | 99.5 |
| **HM324** | PI385014 | Tunisia | M. italica | 25.0 | 41.3 | ~50X KAPA | ~52X Nextera | 132.6 |
| **HM340** | R108-C3 |  | M. tricycla | 33.2 | 45.3 | ~66X TruSeq v3 | ~33X original LIPE, ~58X Nextera LIPE | 157.1 |

^@^SIPE: Short Insert Paired-End library;

^&^LIPE: Lhort Insert Paired-End library;

^*^Coverage was estimated using an assumed genome size of 500 million bases;

^#^KAPA: Kapa biosystems.

Table S2. *De novo* assembly statistics of 15 *M. truncatula* accessions.

|  | Total Span | Total Bases | **Scaffold Stats** | | | | **Contig Stats** | | | | **Repeat Elements** | |
| --- | --- | --- | --- | --- | --- | --- | --- | --- | --- | --- | --- | --- |
|  |  |  | Number | N50 | Median | Max | Number | N50 | Median | Max | Bases | Percent |
| HM101 | 413,771,487 | 389,019,804 |  |  |  |  |  |  |  |  | 88,488,168 | 22.75 |
| HM058 | 409,729,257 | 355,004,629 | 4,349 | 374,919 | 8,122 | 3,222,558 | 27,646 | 18,510 | 4,590 | 350,560 | 66,415,620 | 18.71 |
| HM125 | 406,998,713 | 371,005,289 | 3,666 | 517,348 | 3,690 | 6,709,429 | 22,291 | 28,123 | 5,144 | 417,578 | 77,949,143 | 21.01 |
| HM056 | 406,705,336 | 362,971,816 | 3,486 | 511,115 | 6,755 | 5,985,141 | 26,820 | 19,283 | 5,305 | 230,607 | 71,793,730 | 19.78 |
| HM129 | 398,468,296 | 367,625,895 | 3,213 | 523,031 | 4,777 | 5,629,000 | 21,025 | 28,693 | 5,437 | 303,023 | 74,929,621 | 20.38 |
| HM060 | 403,209,823 | 363,308,695 | 3,634 | 452,558 | 5,845 | 3,832,561 | 22,495 | 25,479 | 5,026 | 396,295 | 72,075,459 | 19.84 |
| HM095 | 410,354,770 | 367,894,112 | 3,711 | 526,756 | 5,178 | 4,466,334 | 24,666 | 25,656 | 4,285 | 323,240 | 73,927,610 | 20.09 |
| HM185 | 428,228,061 | 367,670,036 | 3,335 | 1,653,161 | 3,289 | 16,133,123 | 24,135 | 25,123 | 4,839 | 295,986 | 75,517,964 | 20.54 |
| HM034 | 396,665,787 | 362,786,782 | 3,267 | 471,390 | 4,628 | 6,140,737 | 21,662 | 26,724 | 5,268 | 374,291 | 71,953,536 | 19.83 |
| HM004 | 399,385,294 | 361,118,835 | 3,494 | 620,396 | 3,532 | 9,290,596 | 23,574 | 23,631 | 5,000 | 311,906 | 70,329,693 | 19.48 |
| HM050 | 406,245,560 | 366,441,111 | 3,234 | 855,678 | 3,170 | 8,622,067 | 23,170 | 25,626 | 5,027 | 349,806 | 74,093,668 | 20.22 |
| HM023 | 403,142,311 | 361,243,464 | 3,728 | 421,953 | 7,979 | 5,130,354 | 22,754 | 24,359 | 5,087 | 327,601 | 69,990,997 | 19.38 |
| HM010 | 403,887,869 | 365,935,435 | 3,159 | 522,263 | 6,832 | 6,544,913 | 23,676 | 23,780 | 5,285 | 277,285 | 73,899,843 | 20.19 |
| HM022 | 374,674,840 | 343,656,937 | 2,565 | 694,957 | 5,305 | 8,809,216 | 20,662 | 21,883 | 5,849 | 309,202 | 64,940,061 | 18.90 |
| HM324 | 421,810,942 | 343,081,677 | 7,057 | 267,849 | 6,839 | 4,503,894 | 41,520 | 9,236 | 3,031 | 201,165 | 64,671,095 | 18.85 |
| HM340 | 388,188,365 | 357,565,722 | 3,890 | 674,544 | 2,021 | 7,121,670 | 22,470 | 24,102 | 5,186 | 297,747 | 71,150,565 | 19.90 |

Table S3. Functional annotation statistics of 15 *M. truncatula* accessions.

|  | # Total Genes | TE | non-TE | NBS-LRR | F-box | LRR-RLK | NCR^^^ | Median Prot Length^*^ | RNA-seq (%)^#^ | Homology (%)^&^ | RNA-seq + Homology (%) |
| --- | --- | --- | --- | --- | --- | --- | --- | --- | --- | --- | --- |
| HM101 | 66394 | 19871 | 46523 | 846 | 1172 | 688 | 712 | 255 | 44.7 | - | 44.7 |
| HM058 | 63887 | 16986 | 46901 | 791 | 1087 | 636 | 642 | 245 | - | 86.4 | 86.4 |
| HM056 | 65413 | 18538 | 46875 | 801 | 1111 | 660 | 637 | 245 | 41.6 | 86.4 | 88.5 |
| HM125 | 66976 | 19998 | 46978 | 768 | 1082 | 671 | 642 | 245 | - | 85.9 | 85.9 |
| HM129 | 65416 | 18799 | 46617 | 811 | 1098 | 626 | 630 | 249 | - | 85.1 | 85.1 |
| HM034 | 64342 | 18000 | 46342 | 760 | 1087 | 640 | 630 | 249 | 41.4 | 84.8 | 87 |
| HM095 | 65293 | 18381 | 46912 | 809 | 1097 | 647 | 624 | 246 | - | 84.4 | 84.4 |
| HM060 | 64405 | 17898 | 46507 | 793 | 1085 | 656 | 623 | 248 | - | 85.2 | 85.2 |
| HM185 | 65712 | 19186 | 46526 | 812 | 1070 | 648 | 621 | 248 | - | 84.9 | 84.9 |
| HM004 | 64157 | 17551 | 46606 | 789 | 1083 | 651 | 623 | 250 | - | 84.3 | 84.3 |
| HM050 | 65471 | 18472 | 46999 | 820 | 1090 | 631 | 632 | 248 | - | 84.1 | 84.1 |
| HM023 | 64116 | 17461 | 46655 | 784 | 1091 | 651 | 627 | 248 | - | 84.9 | 84.9 |
| HM010 | 65155 | 18593 | 46562 | 825 | 1080 | 655 | 643 | 249 | - | 85 | 85 |
| HM022 | 59567 | 15223 | 44344 | 700 | 991 | 612 | 647 | 254 | - | 80.5 | 80.5 |
| HM340 | 62188 | 17141 | 45047 | 779 | 981 | 608 | 640 | 252 | 43.1 | 80 | 82.8 |
| HM324 | 60074 | 14922 | 45152 | 732 | 988 | 619 | 606 | 245 | - | 78.4 | 78.4 |

^^^NCR: Nodule cysteine-rich peptides

^*^Median protein length (number of amino acids) was estimated using non-TE coding genes;

^#^RNA-Seq was done for four accessions using both un-inoculated root tissue and nodule; number indicates percentage of total predicted transcripts with FPKM > 0;

^&^Number indicates percentage of total predicted transcripts with at least one Mt4.0 ortholog (either syntenic ortholog or RBH-based homolog).

Table S4. Coverage and diversity statistics by nucleotide class.

|  | **Covered bases (bp)^*^** | | **Total bases (%)** | | | **Polymorphic sites** | | **π bp^-1^** | | **θ_w_ bp^-1^** |  |
| --- | --- | --- | --- | --- | --- | --- | --- | --- | --- | --- | --- |
| **Total** | | 279,689,505 | | - | 7,043,505 | | 0.0073 | | 0.0082 | | |
| **Coding** | | 49,106,309 | | 0.18 | 897,243 | | 0.0052 | | 0.0060 | | |
| **Synonymous** | | 7,219,248 | | 0.03 | 190,416 | | 0.0076 | | 0.0086 | | |
| **Replacement** | | 31,776,906 | | 0.11 | 489,490 | | 0.0044 | | 0.0050 | | |
| **Introns** | | 63,144,752 | | 0.23 | 1,148,781 | | 0.0053 | | 0.0059 | | |
| **UTR 5'** | | 3,505,093 | | 0.01 | 43,689 | | 0.0036 | | 0.0040 | | |
| **UTR 3'** | | 6,241,117 | | 0.02 | 86,575 | | 0.0040 | | 0.0045 | | |
| **Intergenic** | | 157,692,234 | | 0.56 | 4,867,217 | | 0.0089 | | 0.0100 | | |

*Syntenic regions covered by at least 10 (out of 13) in-group accessions

Table S5. Assembly comparison (with Mt4.0) statistics and novel sequences identified in 15 *M. truncatula* accessions

|  | **Total Bases** | **Repetitive** | **Alignable to HM101** | **Bases in Synteny** | **Novel Sequences**^*^ | | **Novel Coding Seq** | |
| --- | --- | --- | --- | --- | --- | --- | --- | --- |
| **HM058** | 355,004,629 | 66,415,620 | 343,325,053 | 323,600,625 | 9,024,826 | 2.50% | 1,297,448 | 14.40% |
| **HM125** | 371,005,289 | 77,949,143 | 357,808,394 | 327,976,297 | 9,863,489 | 2.70% | 1,386,659 | 14.10% |
| **HM056** | 362,971,816 | 71,793,730 | 350,554,159 | 326,377,315 | 9,377,865 | 2.60% | 1,368,844 | 14.60% |
| **HM129** | 367,625,895 | 74,929,621 | 351,879,700 | 320,623,943 | 11,755,160 | 3.20% | 1,631,646 | 13.90% |
| **HM060** | 363,308,695 | 72,075,459 | 347,211,386 | 317,763,091 | 12,044,190 | 3.30% | 1,690,709 | 14.00% |
| **HM095** | 367,894,112 | 73,927,610 | 351,219,964 | 317,035,668 | 12,460,933 | 3.40% | 1,709,362 | 13.70% |
| **HM185** | 367,670,036 | 75,517,964 | 351,105,536 | 317,771,859 | 12,390,758 | 3.40% | 1,729,628 | 14.00% |
| **HM034** | 362,786,782 | 71,953,536 | 346,005,399 | 317,021,576 | 12,433,286 | 3.40% | 1,727,260 | 13.90% |
| **HM004** | 361,118,835 | 70,329,693 | 344,257,187 | 315,304,988 | 12,721,129 | 3.50% | 1,979,711 | 15.60% |
| **HM050** | 366,441,111 | 74,093,668 | 349,114,197 | 317,140,191 | 13,022,686 | 3.60% | 2,157,804 | 16.60% |
| **HM023** | 361,243,464 | 69,990,997 | 344,455,479 | 315,834,571 | 12,376,960 | 3.40% | 1,687,239 | 13.60% |
| **HM010** | 365,935,435 | 73,899,843 | 348,722,480 | 316,833,225 | 12,639,855 | 3.50% | 1,726,239 | 13.70% |
| **HM022** | 343,656,937 | 64,940,061 | 315,921,590 | 275,649,889 | 19,732,777 | 5.70% | 2,099,922 | 10.60% |
| **HM324** | 343,081,677 | 64,671,095 | 312,883,637 | 266,427,488 | 21,757,962 | 6.30% | 2,449,025 | 11.30% |
| **HM340** | 357,565,722 | 71,150,565 | 326,264,086 | 279,190,368 | 20,778,471 | 5.80% | 2,319,870 | 11.20% |

^*^Novel sequences are segments not present in Mt4.0 (HM101) reference.

Table S6. Variants identified in 15 *M. truncatula* accessions by count (A) and affected base pairs (B).

(A)

|  | **SNP^#^** | **SNP Density** | **Small Ins^@^** | **Small Del** | **Large Ins** | **Large Del** | **CNG^*^** | **CNL^$^** | **Translocation** |
| --- | --- | --- | --- | --- | --- | --- | --- | --- | --- |
| HM058 | 1,699,815 | .0057 | 229,566 | 236,818 | 11,716 | 15,100 | 23,266 | 26,208 | 2,720 |
| HM056 | 1,858,188 | .0061 | 247,352 | 255,007 | 12,390 | 15,791 | 26,366 | 27,388 | 3,153 |
| HM125 | 2,075,142 | .0067 | 272,820 | 281,889 | 14,317 | 17,469 | 29,919 | 30,885 | 3,513 |
| HM129 | 2,709,716 | .0091 | 353,523 | 364,363 | 18,667 | 23,139 | 37,076 | 38,803 | 4,953 |
| HM034 | 2,821,493 | .0095 | 368,749 | 377,083 | 19,835 | 25,989 | 37,517 | 39,421 | 5,581 |
| HM095 | 2,722,049 | .0093 | 356,148 | 365,297 | 18,427 | 22,636 | 36,837 | 39,150 | 5,194 |
| HM060 | 2,795,046 | .0094 | 363,601 | 374,109 | 18,602 | 23,586 | 36,516 | 39,458 | 4,942 |
| HM185 | 2,670,644 | .0093 | 348,954 | 359,196 | 18,472 | 22,810 | 36,206 | 38,239 | 4,788 |
| HM004 | 2,860,239 | .0097 | 372,819 | 382,431 | 18,906 | 24,343 | 37,187 | 40,737 | 5,041 |
| HM050 | 2,868,988 | .0097 | 372,123 | 382,998 | 19,498 | 24,529 | 38,145 | 41,014 | 5,225 |
| HM023 | 2,885,692 | .0098 | 375,818 | 385,247 | 19,709 | 26,130 | 37,571 | 39,726 | 5,711 |
| HM010 | 2,906,704 | .0099 | 377,205 | 386,894 | 20,236 | 26,458 | 39,084 | 40,045 | 6,027 |
| HM022 | 5,069,762 | .0206 | 733,635 | 736,313 | 45,302 | 66,834 | 80,780 | 88,265 | 12,456 |
| HM340 | 5,072,373 | .0206 | 723,866 | 735,113 | 45,992 | 64,314 | 82,122 | 86,748 | 12,788 |
| HM324 | 4,984,659 | .0216 | 731,037 | 734,307 | 41,946 | 67,122 | 76,984 | 87,641 | 12,666 |

^#^Numbers listed here are all synteny-based variant calls;

^@^Small Insertions/Deletions are insertions and deletions shorter than 50 base pairs;

^*^CNG: Copy number gain;

^$^CNL: Copy number loss.

(B)

|  | **SNP** | **SNP Density** | **Small Ins** | **Small Del** | **Large Ins** | **Large Del** | **CNG** | **CNL** | **Translocation** |
| --- | --- | --- | --- | --- | --- | --- | --- | --- | --- |
| HM058 | 1,699,815 | .0057 | 1,472,729 | 1,433,967 | 2,907,955 | 4,793,101 | 6,444,318 | 20,368,937 | 3,562,521 |
| HM056 | 1,858,188 | .0061 | 1,604,545 | 1,557,103 | 2,981,372 | 4,857,991 | 8,113,896 | 21,380,128 | 4,226,924 |
| HM125 | 2,075,142 | .0067 | 1,748,580 | 1,717,576 | 3,496,909 | 5,635,199 | 10,027,202 | 24,014,458 | 4,944,633 |
| HM129 | 2,709,716 | .0091 | 2,208,085 | 2,241,253 | 4,614,069 | 7,342,439 | 13,060,326 | 28,844,685 | 6,815,118 |
| HM034 | 2,821,493 | .0095 | 2,363,055 | 2,356,532 | 4,882,635 | 8,945,373 | 12,472,627 | 27,903,777 | 7,324,491 |
| HM095 | 2,722,049 | .0093 | 2,234,135 | 2,242,821 | 4,473,529 | 7,056,774 | 12,803,351 | 28,792,835 | 7,798,090 |
| HM060 | 2,795,046 | .0094 | 2,285,418 | 2,312,236 | 4,494,901 | 7,367,260 | 11,875,765 | 28,864,192 | 6,531,081 |
| HM185 | 2,670,644 | .0093 | 2,210,653 | 2,216,582 | 4,688,974 | 7,036,603 | 11,944,244 | 28,452,857 | 6,883,387 |
| HM004 | 2,860,239 | .0097 | 2,350,976 | 2,375,956 | 4,564,540 | 7,826,327 | 11,743,812 | 29,647,325 | 6,444,519 |
| HM050 | 2,868,988 | .0097 | 2,350,028 | 2,373,850 | 4,705,884 | 7,586,259 | 12,515,726 | 29,768,759 | 6,715,431 |
| HM023 | 2,885,692 | .0098 | 2,425,521 | 2,405,538 | 4,771,137 | 8,801,487 | 11,836,264 | 28,012,528 | 7,383,912 |
| HM010 | 2,906,704 | .0099 | 2,429,096 | 2,423,439 | 4,925,567 | 8,920,031 | 12,921,565 | 28,068,159 | 7,407,099 |
| HM022 | 5,069,762 | .0206 | 5,132,433 | 5,133,963 | 10,211,293 | 21,744,266 | 25,079,221 | 50,472,526 | 13,098,504 |
| HM340 | 5,072,373 | .0206 | 5,079,289 | 5,107,633 | 10,466,002 | 20,071,674 | 27,438,010 | 49,833,972 | 14,307,541 |
| HM324 | 4,984,659 | .0216 | 5,077,977 | 5,325,368 | 9,070,313 | 20,128,282 | 22,585,126 | 46,987,452 | 13,245,864 |

Table S7. SV validation statistics by different SV types and by genomic context.

|  | By SV type | | | | By Genomic Region | | | |
| --- | --- | --- | --- | --- | --- | --- | --- | --- |
|  | Deletion | Insertion | Copy Number Loss | Copy Number Gain | Gene | TE | Unknown | Intergeic |
| HM034 | 93.7% (11,389)^#^ | 94.7% (15,515) | 91.5% (18,840) | 91.3% (18,835) | 92.7% (9,127) | 92.3% (4,159) | 92.7% (45,552) | 91.6% (5,741) |
| HM056 | 90.6% (7,189) | 90.8% (9,262) | 87.1% (14,185) | 86.6% (13,927) | 88.3% (6,578) | 86.7% (2,956) | 88.5% (31,367) | 87.4% (3,662) |
| HM340 | 94.4% (26,249) | 95.0% (36,616) | 93.0% (42,457) | 94.0% (39,783) | 94.2% (30,421) | 94.5% (3,949) | 94.0% (105,245) | 94.5% (5,490) |

^#^Numbers in each cell reflect validation rate (in percentage) followed by total SV number in each class.
