# Supplementary material for: Exploring structural variation and gene family architecture with De Novo assemblies of 15 Medicago genomes
Source: BMC Genomics. 2017 Mar 27;18:261. doi: 10.1186/s12864-017-3654-1 (PMC5369179; doi:10.1186/s12864-017-3654-1)
Supplement: Supplementary file 3 — Supporting data file S1 (Excel spreadsheet listing the member counts of different gene families including all NBS-LRR, NCR, RLK and TE subfamilies, that are predicted in 15 de novo assemblies). (DOCX 940 kb) [file 12864_2017_3654_MOESM3_ESM.docx]

Figure S1. Phylogeny of sequenced *Medicago* accessions with their countries of origin. For more details, see Table S1.

Figure S2. Correlation of assembled genome sizes (ALLPATHS) and fluorometry-based genome size estimates in nine *M. truncatula* accessions.

Figure S3. Tandem duplication of an NBS-LRR in accession HM034 compared with a single copy present in HM101, supported by spanning PacBio reads.

Figure S4A. Distribution of SNP-based nucleotide diversity (i.e., ThetaPi) for different gene families.

Figure S4B. Proportion members affected by different types of large-effect SNPs for different gene families.

Figure S4C. Distribution of mean pairwise protein distance for different gene families.

Figure S4D. Coefficient of variation (C.V.) of gene copy number in each ortholog group among different accessions (i.e., an estimate of copy number variation) for different gene families.

Figure S5. Sharing status of ortholog groups from selected gene families among 13 accessions.


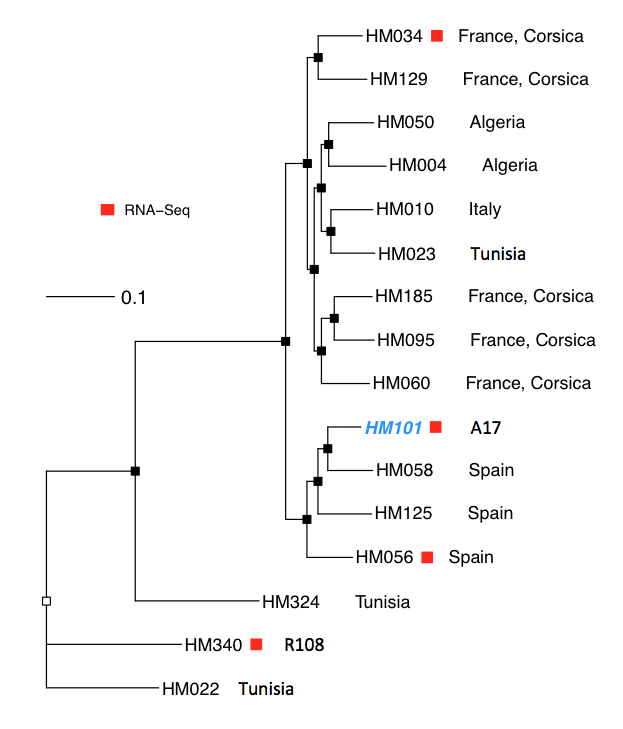


Figure S1. Phylogeny of sequenced *Medicago* accessions with their countries of origin.

Maximum likelihood tree built using 100,000 SNPs randomly sampled from all chromosomes called by the *Medicago* Hapmap project. Nodes with ML bootstrap support of more than 80% are indicated with filled rectangles. Red Rectangles: 4 accessions with RNA-Seq data. Accession HM101 (A17) corresponds to the sequenced reference, Mt4.0.

Figure S2. Correlation of assembled genome sizes (ALLPATHS) and fluorometry-based genome size estimates in nine *M. truncatula* accessions.

Figure S3. Tandem duplication of an NBS-LRR together with a CRP is supported by PacBio reads.

Figure S4A. Distribution of SNP-based nucleotide diversity (i.e., ThetaPi) for different gene families.

Figure S4B. Proportion members affected by different types of large-effect SNPs for different gene families.

Figure S4C. Distribution of mean pairwise protein distance for different gene families.

Figure S4D. Coefficient of variation (C.V.) of gene copy number in each ortholog group among different accessions (i.e., an estimate of copy number variation) for different gene families.

Figure S5. Sharing status of ortholog groups from selected gene families among 13 accessions.
